# What Motivates Medical Students to Engage in Volunteer Behavior During the COVID-19 Outbreak? A Large Cross-Sectional Survey

**DOI:** 10.3389/fpsyg.2020.569765

**Published:** 2021-01-15

**Authors:** Yu Shi, Shu-e Zhang, Lihua Fan, Tao Sun

**Affiliations:** ^1^School of Health Management, Harbin Medical University, Harbin, China; ^2^Department of Health Management and Policy of Hangzhou Normal University, Hangzhou, China

**Keywords:** prosocial motivation, calling and vocation, social responsibility, volunteer behavior, chain mediation model

## Abstract

After the COVID-19 outbreak, the health status of the general population has suffered a huge threat, and the health system has also encountered great challenges. As critical members of human capital in the health sector, medical students with specialized knowledge and skills have positively fought against the epidemic by providing volunteer services that boosted the resilience of the health system. Although volunteer behavior (VB) is associated with individual internal motivation, there is sparse evidence on this relationship among medical students, especially regarding potential mechanisms. Therefore, this study had two main objectives: (1) to examine the influence of prosocial motivation (PM) of medical students on their VB; and (2) to verify the chain-mediating role of calling and vocation (CV) as well as social responsibility (SR) in the relationship between PM and VB. Study I: a total of 2454 Chinese full-time medical students were invited to complete an online survey. Data analysis was performed using descriptive statistics, Pearson’s correlation coefficient, and multiple linear regression analysis. The results demonstrated that PM significantly affected VB in medical students (β = 0.098, *P* < 0.001); CV as well as SR chain-mediated the relationship between PM and VB (β = 0.084, *P* < 0.001). PM promoted the formation of SR by positively evoking CV of medical students, further resulting in increased VB. Study II: A 28 person qualitative interview was conducted. Qualitative data are added to reduce the limitations of online questionnaires. At the same time, we can also critically study the VB of Chinese medical students during COVID-19. The results showed that there were various reasons for medical students to volunteer in the process of fighting against COVID-19, and the experience of volunteer service and the impact on their future life were different. Lastly, the current findings suggest that fostering volunteerism among medical students requires the joint effort of the government, non-profit organizations, and medical colleges.

## Introduction

As a great threat to human life and health (Ali et al., 2020; Wu and McGoogan, 2020), the COVID-19 outbreak worldwide has transmitted rapidly, killing a high number of elderly persons and adults with existing health problems (Gates, 2020). Obviously, global health systems were underprepared for any significant outbreaks despite considerable progress (Bedford et al., 2019). The global prevention and control of COVID-19 has consumed extensive medical resources, leading to a serious shortage of health capital (Quigley et al., 2020). Fortunately, several countries have quickly responded to this dilemma with smart measures. For example, New York University School of Medicine and Columbia University encouraged their medical students to graduate ahead of time to participate in the prevention and control of the epidemic^1^. In fact, a large number of medical students have contributed to the overall process of social prevention and control by participating in various efforts and actions such as case data visualization (Dong et al., 2020), psychological counseling (Jun et al., 2020), translating the latest articles, and compiling epidemiological survey records^2^. As a result, the value of medical students who provide voluntary services during public health emergencies has been recognized for the first time in the field of medical education. Indeed, there is a lack of attention focusing on training aspects of students’ volunteer service ability in traditional medical education. In particular, there is a lack of platforms and channels to support medical students in providing volunteer services. Under the background of public health emergencies, health service workers faced overload work, the challenge of health resources serious scarce, more social participation and support is required (Li et al., 2020), this study focused on the medical students to participate in volunteer service real attitude, motivation and incentive mechanism research, the academic research helps to improve college students volunteer service incentive mechanism.

As a type of long-term free assistance, volunteer behavior (VB) refers to individuals voluntarily responding to those who actively seek help, after careful consideration under special circumstances (Snyder and Omoto, 2010). Although there are many different discussions regarding the definition of VB among scholars, the main characteristics of voluntary service include being voluntary, free, related to public welfare, and altruistic. As a valuable group due to their high professionalization, medical students can play a beneficial role in the field of voluntary service, especially in the area of health promotion (Yan et al., 2014). Therefore, it is of great theoretical and practical significance to encourage medical students to provide volunteer services. Of course, research on medical students’ VB as well as its antecedent factors and functional mechanisms is also necessary.

Numerous empirical studies showed that females, junior students, students with a positive attitude, and students with relevant volunteer service experience are more willing to participate in volunteer service activities (Ueda, 2011). However, in the medical field, we need to break the stereotype of female students and explore the real motivation behind the VB of female medical students, which is also conducive to supplement the theoretical knowledge of voluntary service. More specifically, on a theoretical level, based on a functional perspective of “needs-motivation-behavior” (Omoto and Snyder, 1995), VB has the function of realizing a person’s specific motivation, which explains why individuals tend to participate in volunteer service activities. As an internal force, prosocial motivation (PM) refers to an individual’s desire for action based on the consideration of helping and contributing to others (Grant, 2007), which leads to VB. PM mainly involves a willingness to care for others, build good interpersonal communication, and help others in a crisis (Grant and Sumanth, 2009). In the COVID-19 epidemic, many medical students have actively engaged in voluntary activities, such as providing online consultations, pre-screening and triage, and spreading health knowledge. Thus, we suppose that these initiatives implemented by volunteer medical students may have been inspired by their high PM. In particular, the professional nature of medical students is to cure the disease and save people. The public welfare and dedication are deeply rooted in the learning career of medical students (Kenyon and Brown, 2007). Therefore, we proposed Hypothesis 1 (H1): the PM of medical students has a positive impact on VB.

By integrating various theories, Matsuba et al. (2007) established a multi-factor model in which VB is the result of multiple factors. In their model, influencing factors of VB are divided into persistent and regulatory factors. Persistent factors comprise a series of stable traits including personality, socio-economic and cultural characteristics, and those factors that indirectly affect individual VB. On the contrary, regulatory factors include moral cognition, identity, and opportunity among others.

Moral cognition and identity, as individual internal self-cognition factors, can urge volunteer service directly (Cornelis et al., 2013). Calling and vocation (CV) is a common internal stable trait of medical students that refers to a series of subjective perceptions related to their career role or career expectations, including personal core value, sense of meaning and goal, self-expression, and social contribution (Behavior, 2010). Especially under the guidance of the core idea of “benevolence” in Chinese traditional Confucian culture, medical students with higher CV tend to exhibit a stronger emotional attachment to society by offering medical services (Zhou and Reisach, 2020). When medical staff faces the dilemma of shortage caused by the COVID-19 epidemic, medical students are prone to experience volunteer motivation under the high-powered incentive of CV (Liu shan, 2005). As a consequence, we guess that medical students with high-level CV are more likely to actively assist medical workers to carry out activities of prevention and control of COVID-19.

Calling and vocation has been conceptualized as a guiding force that can boost a series of aspiring career behaviors. Zhang et al. (2015) pointed out that this guiding force includes five elements: sense of responsibility, vision, common expectation, belief, and following destiny. Vision and following destiny both can be seen as external inspiration, while sense of responsibility, belief, and common expectation together emphasize internal drive. As a positive predictor of altruistic behavior (Huang, 2016), sense of responsibility refers to a relatively stable psychological quality by which individuals actively devote themselves to social public service or helping others. Related research indicated that specific social situations can trigger VB (Gebauer et al., 2008), including social context, economy, culture, and other factors. In Eastern cultures, VB is often encouraged by a sense of community, while in countries with individualistic cultures, it is impelled by individuals’ sense of social responsibility (SR) (Snyder and Omoto, 2010). China’s social ethos collide and blend with each other and form tension between each other, which has unprecedentedly aroused public attention and thinking on topics such as democracy and the rule of law, rumors and truth, freedom and equality, individual values and public interests (Jia lizheng and Yanzhuo, 2020). It is possible that higher CV and SR among medical students may have a positive effect on VB during the COVID-19 outbreak. In view of the above, the current study aimed to investigate the functional mechanism between prosocial behavior of medical students and their VB. Therefore, we proposed Hypothesis 2 (H2): medical students’ CV and SR together play a chain-mediating role in the influence of PM on VB.

## Materials and Methods

### Sample/Participants

Study I: A cross-sectional survey was conducted using an anonymous online questionnaire with medical students across 10 Chinese provinces (Henan, Heilongjiang, Liaoning, Hebei, Zhejiang, Jiangsu, etc.). A total of 3,741 participants were recruited. Finally, 2,454 valid questionnaires were used for analysis, yielding an effective response rate of 65.60%. The inclusion criteria for this study included being a full-time college medical student and providing informed consent for voluntary participation. Exclusion criteria included voluntarily choosing not to participate in our study, having an online answering time <4 min, and returning questionnaires with missing items or obvious errors.

Study II: In this study, 28 interviewees were selected in medical colleges, including 23 Medical Students and 5 teachers. There were 12 males and 16 females. The average age was 25.5 years. All respondents have organized or participated in volunteer service. There were 18 students who participated in the volunteer service of the COVID-19 epidemic situation. The specific voluntary service mainly included health knowledge publicity, psychological counseling, community residents registration, and nucleic acid detection.

### Setting

Study I: The anonymous online questionnaire was completed by Chinese medical students throughout the country from April 1 to 19, 2020. With the use of a multistage stratified sampling method was employed, using an open online questionnaire for students from different medical schools (10 medical colleges in China’s central, Eastern and Western Regions). A web page link to our questionnaire survey^3^ was sent via mobile phone to the participants. Students who saw it and were willing to participate could fill it out via mobile phone or computer during their free time. The registered users of the questionnaire (researchers) monitored the collected questionnaires in real time and effectively managed the data using this platform. In the past, our team has successfully used this survey method to complete a series of studies (Zhang et al., 2018; Zhao et al., 2018a,b).

Study II: This study constructs the category and significance of voluntary service motivation according to the research method of root theory (Charmaz, 2000). Semi structured interviews were conducted from October 15 to November 5 to collect data, and collected data in the form of recording and written question and answer questions after obtaining the consent of the interviewee. The core questions of the interview included “How did you participate in volunteer service activities?” “What are the reasons why you participated in the volunteer service for this new outbreak?” In order to find out the interviewees’ experience of participating in volunteer activities, we continued to ask the interviewees’ answers. In the qualitative interview, questions such as “evaluation of the VB” were set up to supplement the purpose of participating in volunteer service and the benefits to be obtained in the end from the perspective of harvest. We converted the recording manuscript into a written manuscript word by word after the interview.

### Ethical Considerations

The study was conducted in compliance with the ethical guidelines of the Ethics Committee of the College of Public Health, Harbin Medical University (HMUIRB20200315), and was approved by the Ethics Committee of the Harbin Medical University. It was not possible to seek written informed consent from the participants because of the anonymous survey approach. However, on the front page of the questionnaire, we clearly clarified that the survey was anonymous. Hence, once a questionnaire was completed and submitted successfully, we assumed the consent of the medical student to participate in our investigation.

### Questionnaire

The survey was divided into two parts: the first part included demographic information for medical students (gender, age, education level, major type, grade, the experience of leadership cadres, registered residence, and family monthly income). The second part includes PM scale, brief calling scale (BCS), student personal responsibility scale, and voluntary behavior scale.

#### Prosocial Motivation

To measure prosocial work motivation, five items developed by Grant (2008) were utilized in the present study. We revised the items’ wording according to the COVID-19 epidemic situation. Participants were instructed to rate the extent to which each of five statements (e.g., *During the period of fighting against the epidemic, I prefer to do some work to increasing the positive influence on others*) was true of them, using a seven-point scale (1 = strongly disagree, 7 = strongly agree). The total score was computed to quantify the respondent’s overall level of PM. The higher the total score, the higher the PM level of medical students. Cronbach’s alpha for this scale was 0.915.

#### Calling and Vocation (CV)

Calling and vocation was evaluated with the BCS, which consists of four items (Dik et al., 2012): “*I still regard my career in medicine and the health industry as a strong pursuit to achieve my life goals even if I know the danger of the epidemic*”; “*I have a good understanding of my calling as it applies to my career*”; “*In the wake of this outbreak, I begin to try to figure out what my mission would be in my future medical career*”; and “*I know the responsibilities of medical workers*. *In my future career, I will go to the front without hesitation if I face the epidemic again*.” Items were scored on a five-point Likert-type scale, ranging from 1 to 5 (1 = not at all true of me, 2 = mildly true of me, 3 = moderately true of me, 4 = mostly true of me, 5 = totally true of me). Thus, higher scores indicated higher CV. Cronbach’s alpha for this scale was 0.898.

#### Social Responsibility

In this study, a SR scale for medical students was developed by referring to the Student Personal Responsibility Scale (SPRS-10) by Singg and Ader (2001). Partial items such as “If my teacher gives me a volunteer task during the epidemic prevention and control period, I will start to complete it immediately,” “During the epidemic, I could think independently and help my family and friends learn scientific knowledge of epidemic prevention to avoid being misled by rumors” and “I believe that COVID-19 prevention and control is the task of countries, health care workers and others.” Participants were requested to rate their SR on a five-point scale (1 = strongly disagree, 5 = strongly agree), with higher scores indicating higher SR. Cronbach’s alpha for this scale was 0.789.

#### Voluntary Behavior

Voluntary behavior was evaluated with four items compiled by Carlo (Carlo et al., 2005). “*In the past, have you participated in voluntary activities?*” “*Are you currently participating in volunteer activities?*” and “*Are you going to take part in voluntary activities in the future?*” were answered as no = 1 or yes = 2; “*In the next year, how likely are you to take part in voluntary activities*?” was answered as a number from 0 to 7, which represented the size of the possibility of volunteering. The higher the total score of these items, the higher the likelihood of voluntary behavior. Erez’s research verified the validity of this questionnaire (Erez et al., 2008).

### Data Analysis

#### Preliminary Analyses

Descriptive statistics were used to inspect the demographic characteristics of participants. The correlations between variables (PM, CV, SR, and VB) were examined using Pearson’s correlation coefficient. All analyses were conducted using SPSS version 25.0 (IBM Corp, BM SPSS Statistics for Windows, Armonk, NY, United States). Statistical significance was defined as a two-tailed *P*-value < 0.05.

#### Chain-Mediation Analysis

A hierarchical regression analysis was conducted to test the chain-mediation effects. The chain intermediary mechanism was investigated with the SPSS macro PROCESS provided by Preacher and Hayes (Hayes, 2013a), and Model 6 was executed. The chain intermediary analyses were based on bootstrapping (5,000 bootstrap samples) using 95% confidence intervals (CIs). The macro PROCESS was used for calculating and testing the direct and indirect effects. The mediation mechanism is significant when the 95% CI does not include 0. Gender, age, education level, grade, major type, family monthly income, and experience of leadership cadre among medical students were included as control variables (Singg and Ader, 2001; Matsuba et al., 2007).

#### Analysis of Interview Data

Step 1: we encode all the text materials with words as the basic unit. That is to say, taking words as the basic unit, not omitting any important information, using the original words and sentences of interviewees as the basis of concept classification as far as possible, and comparing the open codes repeatedly until they are saturated. Step 2: we discover and establish various generic relationships among conceptual genera in the process of relational coding login. While analyzing the relevance of conceptual categories and exploring the relationship between them, we also explore the volunteer intention and motivation of the interviewees, and put their words into the context of the time and their social and cultural background. Step 3: We analyzed the correlation coding in the previous step and carried out the generic analysis. The core code occupies the central position of all genera and becomes the core of the data. It is easy to connect with other genera and is easy to be summarized as theory. This method of analyzing qualitative data has been widely used in numerous studies (Howell, 2009; Mulholland et al., 2019).

## Results

Participants’ demographic characteristics are shown in Table 1. The majority of the sample was female (74.8%). Participants aged 20–21 accounted for half of the sample (55.22%). Almost all participants were undergraduates (91.93%), with a clinical medicine major (21.1%). Third-year undergraduate students were the most numerous (31.8%), compared with other grades. Participants who had experience of leadership cadre among medical students (51.5%) were slightly more numerous than those with no experience (48.5%). Furthermore, the number of participants was basically the same for urban areas (45.8%) and rural areas (54.2%). Moreover, 38.4% had a family monthly income within 2,001–5,000 RMB.

**TABLE 1:** Characteristics of participants (*n* = 2,454).

Characteristic	Classes	*N*	%
Gender	Male	618	25.2
	Female	1836	74.8
Age	16–17	4	0.20
	18–19	477	19.44
	20–21	1355	55.22
	22–23	529	21.56
	24–25	73	2.97
	26–27	12	0.49
	28–29	4	0.20
Education level	Specialist stage	179	7.29
	Undergraduate	2256	91.93
	Post-graduate and above	19	0.78
Major type	Clinical medicine	518	21.1
	Anesthesiology	63	2.60
	Stomatology	32	1.30
	Imaging medicine	449	18.3
	Nursing	344	14.0
	Pharmacy or clinical pharmacy	153	6.20
	Preventive Medicine	43	1.80
	Health Care Management	329	13.4
	Basic medicine	29	1.20
	Other majors	494	20.1
Grade	First year in college	691	28.2
	Second year in college	766	31.2
	Third year in college	780	31.8
	Fourth year in college	138	5.6
	Fifth year in college	79	3.2
The experience of leadership cadres	Experience	1264	51.5
	No-experience	1190	48.5
Registered residence	Rural area	1331	54.2
	Urban area	1123	45.8
Family monthly income	≤2000 RMB	363	14.8
	2001–5000 RMB	943	38.4
	5001–8000 RMB	566	23.1
	8001–10000 RMB	323	13.2
	10001–20000 RMB	200	8.1
	≥20001 RMB	59	2.4

Score means and SDs, and internal consistencies for all the measures were computed. Pearson’s correlation coefficients for continuous variables are shown in Table 2. All variables were significantly correlated with each other. PM was positively correlated with CV (*r* = 0.589, *P* < 0.01), SR (*r* = 0.591, *P* < 0.01), and VB (*r* = 0.445, *P* < 0.01). Moreover, CV was positively correlated with SR (*r* = 0.553, *P* < 0.01) and VB (*r* = 0.430, *P* < 0.01). Moreover, there was a positive correlation between SR and VB (*r* = 0.389, *P* < 0.01).

**TABLE 2:** Mean, SD, and internal consistency of study variables.

	Variables	*M*	SD	α	1	2	3	4
1	Prosocial motivation	3.607	0.460	*0.915*	*–*			
2	Calling and Vocation	4.160	0.864	*0.898*	0.589**	–		
3	Social responsibility	4.811	1.113	0.798	0.591**	0.553**	–	
4	Volunteer behavior	3.150	0.581	–	0.445**	0.430**	0.389**	–

Italics represent the internal consistency coefficient of the scale. **p < 0.01; correlation is significant at the 0.01 level (2-tailed).

**FIGURE 1:**
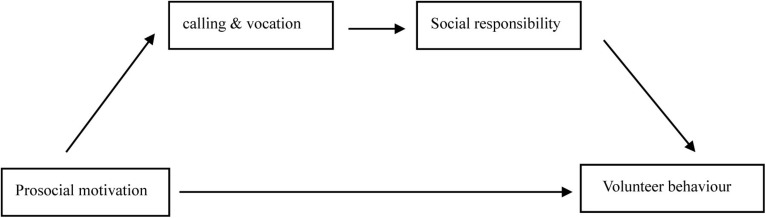
The chain-mediation model.

The results of the chain-mediation analysis are summarized in Table 3. Model 6 in the model library developed by Hayes (2013b) was executed with the SPSS macro PROCESS. First, in this analysis, gender, age, education level, grade, major type, family monthly income, and experience of leadership cadre were treated as control variables in the regression equations. A model was constructed with CV (M1) as a mediator and SR (M2) as another mediator. In this model, PM was set as the predictor (X) and VB as the outcome (Y). We used 5,000 bootstrap samples and determined the mediating effect using 95% CIs. The results showed that X had a significant positive predictive effect on Y (β = 0.098, *P* < 0.001), and H1 was thus supported. Moreover, the indirect effect of X on Y was significant (lower limit CI = 0.069, upper limit CI = 0.099). Therefore, CV and SR played a chain-mediating role in the relationship between PM (X) and VB intention (Y) among medical students; H2 was thus supported. The chain-mediation model is shown in Figure 1.

**TABLE 3:** Regression model of the effect of prosocial motivation on volunteer behavior among Chinese medical students.

Item	Model 6 in Model Templates						
	R^2^	Coefficient	SE	*t*	*P*	LLCI	ULCI
**Outcome variable: C and V**							
Constant	0.368	89.222	101.732	0.877	0.381	–110.268	288.712
Independent variable: PM		0.427	0.012	35.6	<0.001	0.404	0.451
**Control variable**							
Gender		–0.11	0.123	–0.972	0.331	–0.331	0.112
Age		–0.042	0.051	–0.832	0.405	–0.142	0.057
Education level		–0.186	0.179	–1.041	0.298	–0.538	0.165
Grade		–0.267	0.7	–3.831	<0.001	–0.404	–0.13
Professional type		–0.106	0.016	–6.846	<0.001	–0.137	–0.076
Family monthly income		0.031	0.4	0.790	0.43	–0.047	0.109
The situation of student cadres		–0.193	0.054	–3.570	<0.001	–0.299	–0.087
**Outcome variable: SR**							
**Constant**	0.422	95.395	153.298	0.622	0.534	–205.212	396.002
M 1 variable: C and V		0.509	0.031	16.606	<0.001	0.449	0.569
Independent variable: PM		0.471	0.022	21.089	<0.001	0.427	0.514
**Control variable**							
Gender		–0.228	0.170	–1.339	0.181	–0.561	0.106
Age		–0.038	0.077	–0.502	0.616	–0.189	0.112
Education level		–0.623	0.270	–2.308	0.021	–1.152	–0.094
Grade		0.186	0.105	1.766	0.076	–0.021	0.393
Professional type		0.083	0.024	3.519	<0.001	0.037	0.130
Family monthly income		0.224	0.060	3.739	<0.001	0.107	0.341
The situation of student cadres		–0.016	0.082	–0.201	0.840	–0.177	0.144
**Outcome variable: VB**							
**Constant**	0.268	93.148	63.251	1.423	0.141	–30.883	217.178
M 1 variable: C and V		0.121	0.013	9.098	<0.001	0.095	0.147
M 2 variable: SR		0.047	0.008	5.683	<0.001	0.031	0.064
Independent variable: PM		0.098	0.010	9.817	<0.001	0.079	0.118
**Control variable**							
Gender		0.121	0.072	1.718	0.860	–0.017	0.258
Age		–0.045	0.032	–1.417	0.157	–0.107	0.017
Education level		–0.054	0.111	–0.488	0.626	–0.273	0.164
Grade		–0.034	0.044	–0.789	0.430	–0.120	0.051
Professional type		0.003	0.010	–0.289	0.773	–0.022	0.016
Family monthly income		–0.700	0.025	–2.814	0.004	–0.118	–0.021
The situation of student cadres		–0.236	0.034	–7.014	<0.001	–0.302	–0.170
		Effect	Boot SE			Boot LLCI	Boot ULCI
Indirect effect(s) of PM on VB	–	0.084	0.008	–	–	0.069	0.099

*PM, prosocial motivation; C and V, calling and vocation; SR, social responsibility; VB, volunteer behavior.*

According to the three-level coding analysis (Table 4), this study found that the generation of volunteer service behavior is affected by many factors. It mainly includes the motivation of volunteers and volunteer network. The motivation mainly includes altruistic motivation, PM, self-moral cognition and incentive factors. The voluntary network mainly includes school, social and network channels. The 18 students who participated in the prevention and control of COVID-19 volunteers had different experiences, including positive volunteer service experience and negative volunteer service experience, as well as the negative impact of these experiences on medical students.

**TABLE 4:** Code table of medical students’ volunteer behavior research during COVID-19.

Open coding	Selective coding	Core coding
Help medical staff reduce stress, Promote health knowledge	Altruistic Motivation	Volunteer motivation
Refute rumors, community service, Gate registration, Online tutoring for children of medical staff	Prosocial motivation	
Logistics support, Disinfection work, psychological counseling	Self-moral cognition	
Share national pressure, Sense of meaning, Sense of responsibility, professional advantage	Incentive factors	
Exercise ability, enrich spare time, broaden vision, obtain social recognition		
Professional recognition, honorary certificate, volunteer service photo souvenir, volunteer service written proof, expand the interpersonal communication network		
Class, volunteer association, community, student union, research team	School	Volunteer Network
Community health service center, family and friends, self-contact, social recruitment	Society	
WeChat group self-organization, WeChat official account, Internet News	Information network	
Meaningful, valuable, harvest, exercise their own practical ability, sense of achievement	Positive experience	Volunteer experience
Practice their own medical student oath, increased the team cohesion	Negative experience	
Formalism, lack of mature organization and discipline, vague work role		
Unreasonable working time arrangement, lack of professional training, heavy workload		
Self-value realization, Sense of professional mission	Positive impact	Volunteer experience follow-up impact
Self-enrichment during home isolation		
There is a big gap between the actual work and the expected work, Policy coordination	Negative influence	
Waste of time, interpersonal communication barriers, work maladjustment		

## Discussion

### Medical Students With High Prosocial Motivation Are More Likely to Engage in Volunteer Behavior During Public Health Emergencies

We found that medical students with high PM engaged in more frequent VB during the COVID-19 outbreak, which is similar to the results of a previous study conducted among college students in the United States (Carlo et al., 2005). A survey on volunteers with a long-term follow-up by Snyder also supported the same viewpoint that individual motivation directly affects VB (Gebauer et al., 2008), most of the students with high PM will continue to participate in volunteer activities in the future, even if the previous volunteer service experience is not perfect, the results of this qualitative interview are consistent with the above research. All respondents said that they will still participate in different types of volunteer services in the future, once there is a suitable time and opportunity. Meanwhile, Grant argued that individual PM regarded as a kind of current psychological state is usually aroused by the desire to help others, thereby leading individuals to focus on their internal motivation to protect and improve the welfare of others (Grant, 2008). Because individual PM can be stimulated in a disaster scenario, medical students will consider how they can contribute to fighting against the epidemic. For example, Some college students in China have volunteered to serve as tutors for the children of medical workers in order to help medical workers relieve part of their family burden. Although these students do not contribute directly to the medical field, they also indirectly help health care workers to have more energy to fight the COVID-19 epidemic. In the qualitative interview, we found that female students are more likely to expand their interpersonal network and practice ability by participating in volunteer service, so as to make their future career more smooth.

Previous studies have divided PM of employees into three levels based on the basic framework of psychological motivation research (Clary et al., 1998; Grant and Berry, 2011; Hu et al., 2013). First, global PM, as a relatively stable personality tendency, refers to employees with high-degree intrinsically prosocial values who tend to attach importance to protecting and increasing the overall welfare of others. Second, contextual PM refers to employees who want to help specific groups in a unique situation (natural disasters, infectious diseases, etc.). For instance, employees resort to their professional roles to help others during work. In the stage of fighting COVID-19, Chinese virtues such as fearless sacrifice, courage to take responsibility, selfless dedication, solidarity and mutual assistance have been fully demonstrated and carried forward, reflecting that the spiritual quality of the whole nation has risen to a new level (Anlong, 2020). Third, situational PM represents a more specific and dynamic trait (Berg and Grant, 2012); For example, doctors and nurses perform duties during major public health emergencies. To sum up, we can assume that both contextual and situational PM lead medical students to engage in prosocial behavior after considering the containment practices for the COVID-19 epidemic. Considering the suggestion that volunteer experiences are prone to trigger further realization of individuals’ initial motivation (Hu et al., 2013), medical students with satisfactory volunteer experiences will be significantly more likely to engage in VB in the future (Clary et al., 1998). Therefore, educators in medical colleges need to pay attention to the management of students’ volunteer service by building a continuous volunteer service system, so as to provide a platform for further promoting medical students’ PM.

### CV and Social Responsibility Play a Chain-Mediating Role in the Influence of Prosocial Motivation on Volunteer Behavior

The current study verified a chain-mediating effect of the CV and SR of medical students on the relationship between PM and VB, consistent with previous scholars’ conclusions (Matsuba et al., 2007). Of course, a series of antecedents as essential conditions for triggering individual VB are inevitable, while the process variables determine their trend of occurrence and development (Jaffe et al., 2010; Liu and Zhao, 2016; Shi et al., 2017). Matsuba suggested that the intermediary factors in the relationship between antecedents and VB mainly included individual moral cognition, self-identity, and opportunity (Matsuba et al., 2007). Snyder posed that volunteering is not only a behavior but also a part of identity that reflects the goals and significance of many people’s lives (Snyder, 2001). The results of qualitative interviews supplement this view, the identity of medical students makes medical students have empathy for the working state of medical staff. They are more willing to help medical staff and society to contribute their “strength.” Whether it is medical related work or other public services, their inner PM and SR drive them to actively participate in volunteer service. According to the role identity model, Grube (2000) suggested that individual role identity was a determinant factor in the process of VB; in particular, the factors of self-recognition and identity in volunteer activities were described as important components of self-identity. More specifically, if individuals realize the value of their volunteer work, they tend to be satisfied with the experience or regard “volunteering” as a part of their identity, which leads to them becoming highly integrated with volunteer service organizations (Penner et al., 2005). In addition, respondents also proposed medical students’ CV for a career in medicine increases their sense of identity as health care providers after undergoing their training for professional knowledge and practice skills.

When the whole society urgently needs medical workers to participate in containing the epidemic, as a motivator, this situation is likely to arouse medical students’ sense of SR, PM, and altruism by increasing their CV to maintain the safety and health of the population. It is worth mentioning that the written proof of voluntary service and interpersonal network are also the incentive factors to encourage medical students to participate in voluntary service. The Hippocratic oath (Sher, 1996), as the value driver for medical students, often inspires them to engage in voluntary service and help people to avoid the COVID-19 threat. For instance, the College Student Volunteer and Medical Staff Family Pairing Program initiated by Chinese college students established a service team during the epidemic to help the offspring of medical staff with coaching lessons. The interviewed medical students and teachers also said that participating in volunteer activities is conducive to improving medical students’ self-identity and quality education in schools. Moreover, medical students in different regions contribute to various kinds of services, such as interpretation of national epidemic prevention policies, helping the poor, providing psychological counseling, and organizing donations. These voluntary behaviors not only exhibit the expertise of medical students but also cultivate their practical ability to serve society. Therefore, educators in medical colleges need to attach importance to fostering the enthusiasm of medical students’ for volunteering. Meanwhile, policymakers in the government and public organizations need to positively create various platforms for providing more job opportunities to motivate medical students’ volunteer services.

This study explained the functional mechanism between PM in medical students and their VB, specifically indicating that CV and SR play a mediating role in this link. Findings from research on the chain-mediating effect also highlighted the process of generation and reinforcement of VB of medical students (liang, 2013), further clarifying the role of PM in the mechanism of voluntary service. In sum, our findings highlight that medical education organizations should pay attention to cultivating students’ sense of SR and offer more job opportunities for medical students to contribute to volunteer services during their study period. Besides, volunteering in the medical service industry must be paid enough attention at both academic and practical levels in the future.

### Limitations

Although the present study yielded significant results, several limitations must be mentioned. First, the non-random sampling network survey method potentially causes sample bias, which can affect the study results. The questionnaire distribution method used in this study may also lead to potential but incalculable sample size bias. Second, the cross-sectional nature prevented the establishment of a causal relationship between the variables. Therefore, one important suggestion is that longitudinal studies should be conducted in the future. Third, data were self-reported, which may lead to potential errors caused by memory bias. What’s more, this study uses a combination of qualitative and quantitative research to minimize the bias of the research results, but some subtle issues such as social approval still have certain limitations on the research results. There are also issues of political correctness and need of acceptance in the medical field. These limitations need to be addressed in future research.

## Conclusion

In order to avoid the risk of infection involved in face-to-face surveys, a total of 2,454 medical students were surveyed using an online questionnaire. Data on PM, CV, SR, and VB were collected through self-report. Qualitative interviews were also carried out after the opening of Chinese schools. A total of 28 teachers and students participated. The results indicated that greater PM in medical students can positively predict more frequent VB. Moreover, CV and SR played a chain-mediating role in the relationship between PM and VB. In other words, medical students with high PM will have greater SR by increasing their CV, which further triggers subsequent VB. The results of the qualitative interview further enriched the research content of the questionnaire, and explored the deeper motivation and feelings of medical students to participate in volunteer activities. Finally, we propose that governments, non-profit organizations, public welfare organizations, and medical colleges need to pay more attention to foster students’ CV and SR. All-level organizations should contribute to jointly provide rich job opportunities and platforms for generating volunteer services for medical students, and need to build a sustainable incentive system to encourage medical students to engage in VBs to serve society.

## Data Availability Statement

The raw data supporting the conclusions of this article will be made available by the authors, without undue reservation.

## Ethics Statement

The study was conducted in compliance with the ethical guidelines of the Ethics Committee of the College of Public Health, Harbin Medical University (HMUIRB20200315), and was approved by the Ethics Committee of the Harbin Medical University. It was not possible to seek written informed consent from the participants because of the anonymous survey approach. However, on the front page of the questionnaire, we clearly clarified that the survey was anonymous. Hence, once a questionnaire was completed and submitted successfully, we assumed the consent of the medical student to participate in our investigation.

## Author Contributions

YS conceptualized and designed the study with assistance from LH F and TS. YS and S-E Z performed the quantitative analysis. YS wrote the manuscript, recruited the survey respondents. All authors have reviewed and approved the manuscript prior to submission.

## Conflict of Interest

The authors declare that the research was conducted in the absence of any commercial or financial relationships that could be construed as a potential conflict of interest.

## Footnotes

1 https://www.medscape.com/public/univadis/medscape-news

2 https://www.zyz.org.cn/

3 https://www.lediaocha.com/survey

## Abbreviations

PM: prosocial motivation
CV: calling and vocation
SR: social responsibility
VB: volunteer behavior.
